# The Characterization of High-Fat Diet and Multiple Low-Dose Streptozotocin Induced Type 2 Diabetes Rat Model 

**DOI:** 10.1155/2008/704045

**Published:** 2009-01-04

**Authors:** Ming Zhang, Xiao-Yan Lv, Jing Li, Zhi-Gang Xu, Li Chen

**Affiliations:** Department of Pharmacology, School of Basic Medical Sciences, Jilin University, Changchun, Jilin 130021, China

## Abstract

*Aim*. Based on the previously established method, we developed a better and stable animal model of type 2 diabetes mellitus by high-fat diet combined with multiple low-dose STZ injections. Meanwhile, this new model was used to evaluate the antidiabetic effect of berberine. *Method*. Wistar male rats fed with regular chow for 4 weeks received vehicle (control groups), rats fed with high-fat diet for 4 weeks received different amounts of STZ once or twice by intraperitoneal injection (diabetic model groups), and diabetic rats were treated with berberine (100 mg/kg, berberine treatment group). Intraperitoneal glucose tolerance test and insulin tolerance test were carried out. Moreover, fasting blood glucose, fasting insulin, total cholesterol, and triglyceride were measured to evaluate the dynamic blood sugar and lipid metabolism. *Result*. The highest successful rate (100%) was observed in rats treated with a single injection of 45 mg/kg STZ, but the plasma insulin level of this particular group was significantly decreased, and ISI has no difference compared to control group. The successful rate of 30 mg/kg STZ twice injection group was significantly high (85%) and the rats in this group presented a typical characteristic of T2DM as insulin resistance, hyperglycemia, and blood lipid disorder. All these symptoms observed in the 30 mg/kg STZ twice injection group were recovered by the treatment of berberine. *Conclusion*. Together, these results indicated that high-fat diet combined with multiple low doses of STZ (30 mg/kg at weekly intervals for 2 weeks) proved to be a better way for developing a stable animal model of type 2 diabetes, and this new model may be suitable for pharmaceutical screening.

## 1. INTRODUCTION

Now there has been a tragic increase in diabetes across the world, paralleling the overweight and obesity epidemic. There are 95 percent of those people belonging to type 2 diabetes. Therefore, it is great urgency to find better treatments and novel prevention strategies for type 2 diabetes. To accomplish this goal, appropriate experimental models are considered as essential tools for understanding the molecular basis, pathogenesis of the vascular and neural lesions, actions of therapeutic agents, and genetic or environmental influences that increase the risks of type 2 diabetes.

Although there are numerous animal models (natural as well as developed) available for the study of type 2 diabetes [1–4], the pattern of disease establishment and progress in most of them did not appear to be similar to the clinical situation in humans. Thus, there is a continued quest among the investigators with respect to the establishment of better animal model for type 2 diabetes by adjusting the existing methods, developing new methodologies, or a combination of both.

Many studies have reported that the rats fed with high-fat diet (HFD) develop insulin resistance but not frank hyperglycemia or diabetes [5–7]. It is suggested that the HFD might be a better way to initiate the insulin resistance which is one of the important features of type 2 diabetes. At the same time, streptozotocin (STZ) is widely used to reproducibly induce both insulin-dependent and noninsulin-dependent diabetes mellitus presently by inducing *β* cell death through alkylation of DNA [8]. Although high-dose STZ severely impairs insulin secretion mimicking type 1 diabetes, low-dose STZ has been known to induce a mild impairment of insulin secretion which is similar to the feature of the later stage of type 2 diabetes [1, 2]. Therefore, investigators have started to develop a rat model by feeding the animal with high-fat diet following low-dose STZ that would closely mimic the natural history of the disease events (from insulin resistance to *β* cell dysfunction) as well as metabolic characteristics of human type 2 diabetes [1, 2, 4]. The successful establishment of such a model would be cheaper, easily accessible, and practical for the investigation as well as testing of various compounds for the treatment of type 2 diabetes. Although the appearance of the type 2 diabetes pattern was achieved by combining the feeding of HFD and low dose of STZ treatments in nongenetic, out-bred rats, the injection dose of STZ and its methodologies were not consistent in those studies. Others reported that STZ may also be given in multiple low doses. It has been extensively used in the development of type 1 diabetes in rats and mice to study immune response in pancreas, since the multiple low-dose injections of STZ could induce a gradual, autoimmune destruction of *β* cells instead of the rapid destruction induced by a single high-dose injection [9–14]. However, it has not been reported whether the high-fat diet has synergistic effect on accelerating the development of type 2 diabetes with multiple low doses of STZ.

The purpose of the present study is to develop an appropriate, stable animal model which is analogous to the human type 2 diabetes mellitus through a combination of high-fat diet with multiple low-dose STZ injections. As a result, we provide a suitable animal model to understand the possible cellular and molecular mechanisms of type 2 diabetes. Meanwhile, the treatment has been conducted. Berberine (C_20_H_18_C_1_NO_4_) is the major active constituent of *Rhizoma coptidis*, chemically named 5,6-Dihydro-9,10-dimethoxybenzo(g)-1,3-dioxolobenzo(5,6a) quinolizine chloride. It was commonly used to treat diarrhea as an antimicrobial agent before. Recently, it has been demonstrated that berberine is available for the treatment of diabetes patients [12–15]. In the present study, we will provide berberine to this model to evaluate whether it could cure the diabetes induced by high-fat diet combined with new models of multiple low-dose STZ injections.

## 2. MATERIALS AND METHODS

### 2.1. Materials

STZ was purchased from Sigma, insulin was purchased from Eli Lilly, Changchun, China; glucose, total cholesterol (TC), and triglyceride (TG) test kits were obtained from Beijing BHKT Clinical Reagent Co., Ltd, Beijing, China; iodine [^125^I] insulin radioimmunoassay kit was purchased from Tianjing Nine Tripods Medical & Bioengineering Co., Ltd, Tianjing, China; Other reagents were purchased from Beijing General Chemical Reagent Factory, Beijing, China. Berberine was a gift from Northeast drug factory.

### 2.2. Experimental protocol

Male Wistar rats (200–250 g) were purchased from the Experimental Animal Holding of Jilin University. The animals were housed in standard polypropylene cages (three rats/cage) and maintained under controlled room temperature and humidity with 12/12-hour light-dark cycle. Regular chow consisting of 5% fat, 53% carbohydrate, 23% protein, with total calorific value 25 kJ/kg and high-fat diet consisting of 22% fat, 48% carbohydrate, and 20% protein with total calorific value 44.3 kJ/kg were ordered from the stoyer center of Experimental Animal Holding. Experiments were conducted in the following three sections.

#### 2.2.1. First section

100 Wistar rats were randomly divided into 5 groups: control group (CON1), model group 1 (DM1), model group 2 (DM2), model group 3 (DM3), and model group 4 (DM4); control group was fed with regular chow, and other four groups were given high-fat diet for 4 weeks; four model groups were injected intraperitoneally (IP) with different doses of STZ (STZ was injected only once, DM1: 25 mg/kg; DM2: 30 mg/kg; DM3: 35 mg/kg; DM4: 45 mg/kg), while the control rats were given vehicle citrate buffer (pH 4.4) in a dose volume of 0.25 mL/kg IP, respectively. The body weight and food intake were recorded every week. After 8 weeks of STZ injection, all the rats were fasted for 12 hours; the fasting blood glucose (FBG) analysis was carried out. The successful rate was calculated. The fasting blood insulin (FINS) was measured; intraperitoneal glucose tolerance test (IPGTT) and insulin tolerance test (ITT) were carried out in control and highest successful rate model group.

#### 2.2.2. Second section

60 Wistar rats were randomly divided into 3 groups: control group (CON2), model group 5 (DM5), and model group 6 (DM6); control group was fed with regular chow, and other two groups were given high-fat diet for 4 weeks; two model groups were injected IP with a low dose of STZ (STZ was injected twice, DM5: 25 mg/kg; DM6: 30 mg/kg). After one week, FBG was measured, the rats with FBG < 7.8 mmol/L were injected with STZ again (DM5: 25 mg/kg; DM6: 30 mg/kg), while the control rats were given vehicle citrate buffer (pH 4.4) in a dose volume of 0.25 mL/kg, IP, respectively. The body weight and food intake were recorded every week. After 8 weeks of STZ injection, all the rats were fasted for 12 hours, FBG was carried out. The successful rate was calculated. Intraperitoneal glucose tolerance test (IPGTT) and insulin tolerance test (ITT) were carried out in control and highest successful rate model group.

#### 2.2.3. Third section

100 Wistar rats were randomly divided into 5 groups: control group (CON3), control plus STZ injection group (C-STZ), high-fat diet group (HFD), high-fat diet plus STZ injection group (HFD-STZ), Berberine treated high-fat diet plus STZ injection group (BER); control group and control plus STZ injection group were fed with regular chow, and other three groups were given high-fat diet for 4 weeks; the C-STZ, HFD-STZ groups and BER group were injected IP with a low dose of STZ (according to the second section, choosing the dose of the group with higher successful rate: 30 mg/kg). After one week, FBG was measured in these three groups, the rats with FBG < 7.8 mmol/L were injected with STZ again (30 mg/kg), while the control rats were given vehicle citrate buffer (pH 4.4) in a dose volume of 0.25 mL/kg, IP, respectively. The fasting blood glucose was measured every week. After 4 weeks of STZ injection, the rats with the fasting blood glucose of ≥7.8 mmol/L twice or with nonfasting blood glucose of ≥11.1 mmol/L were considered diabetic. Berberine (100 mg/kg body weight) was administered orally as suspension by mixing with vehicle 1% Na-CMC at a dose volume of 0.5 mL/kg body weight of rats in treatment group for another 4 weeks. The body weight and food intake of the animals were also measured. The rats were allowed to continue to feed on their respective diets until the end of the study. At the end of the study, IPGTT and ITT were conducted in the five groups; fasting plasma was collected for further measurement of insulin, TG, TC, and glucose. The insulin sensitivity index (ISI) was calculated according to the fasting insulin and glucose concentration.

### 2.3. Measurement of FBG, FINS, TG, TC

Rats were fasted for 12–16 hours. Blood was collected from tail vein; plasma was separated by centrifuge at 3500 × g for 10 minutes. Fasting blood glucose (GOD-POD, glucose oxidase-peroxidase), TC (CHOD-POD, cholesterol oxidase peroxidase), and TG (GPO-POD, glycerol-phosphoric acid oxidase peroxidase) were measured by using commercially available colorimetric diagnostic kits according to the instruction. Plasma insulin was assayed by RIA according to the instruction.

### 2.4. Diabetic model successful rate and insulin sensitivity index

Diabetic model successful rate referred to the percentage of diabetic rats in the group. The glucose level and insulin level of the same rat were measured and its insulin sensitivity index (ISI) was calculated as Ln(FBG × FINS)^−1^.

### 2.5. Intraperitoneal glucose tolerance test

After an overnight fast (12–16 hours), the rats were IP injected with 40% glucose (2 g/kg body weight). Blood samples were collected from the tail at 0, 30, 60, and 120 minutes for measurement of glucose.

### 2.6. Insulin tolerance test

Insulin (0.75 IU/kg) was administered by intraperitoneal injection and blood samples were collected at 0, 30, 60, and 120 minutes for the measurement of plasma glucose. The value is presented as a percentage of initial plasma glucose level.

### 2.7. Statistical analysis

The data are reported as mean ± SEM (*n* = 17–20/group). Statistical analysis was performed by one way ANOVA. *P* < .05 was considered a statistical significance between control and experimental groups.

## 3. RESULTS

### 3.1. FBG and diabetic successful rate in the groups of the first section

Table 1 illustrated the diabetic successful rate (DSR) and FBG level in rats which were fed with high-fat diet combined with STZ 25 mg/kg, 30 mg/kg, 35 mg/kg, and 45 mg/kg. As demonstrated in Table 1, the successful rate in 25 mg/kg, 30 mg/kg, and 35 mg/kg STZ injection groups was low (10%, 35%, and 40%, resp.). However, 45 mg/kg STZ injection had the highest successful rate (100%), its FBG level was also significantly high compared to control group (*P* < .001).

### 3.2. IPGTT, ITT, and ISI in the groups of the first section

**Figure 1:**
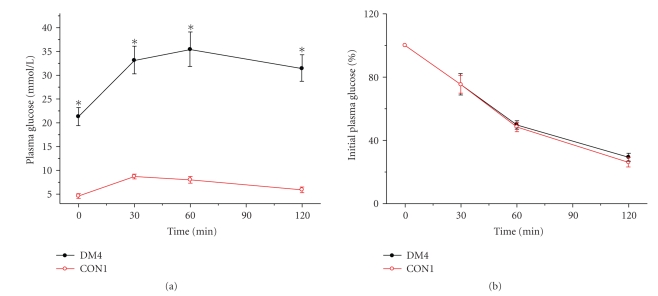
(a) Plasma glucose during intraperitoneal glucose tolerance test (IPGTT) in STZ (45 mg/kg, IP) group (DM4) and control rats after 8 weeks of injection. (b) Percentage of initial glucose level during insulin tolerance test (ITT) in DM4 and control groups after 8 weeks of injection. Data shown are means ± SE (*n* = 20 rats/group per time point). *P < .001, DM3 versus control, by *t*-test.

Our data showed that 45 mg/kg STZ injection (DM4) had the highest successful rate and higher FBG level. Therefore, IPGTT and ITT were carried out in DM4 and control groups to measure glucose tolerance and insulin sensitivity. As shown in Figure 1(a), DM4 showed hyperglycemia compared to control rats during 120 minutes after glucose injection. The areas under the glucose curves (mmol/L·min) were significantly greater in the DM4 group compared with controls [(4000 ± 153) mmol/L·min versus (967 ± 46) mmol/L·min, *P* < .001]. To investigate differences in insulin sensitivity, we performed an ITT at different time points (Figure 1(b)). Insulin was given intraperitoneally and blood was collected for the measurement of glucose. In control group, glucose concentrations declined rapidly after insulin administration, and the decrease became significant by 30 minutes. However, there was no significant difference between the STZ 45 mg/kg injection group and control groups during ITT. This result demonstrated that the rats after STZ 45 mg/kg injection were sensitive to insulin; the ISI also supported this point (Table 2). All the data indicated that high-fat diet associated with 45 mg/kg STZ injection developed a diabetic model which was prone to type 1 diabetes mellitus.

### 3.3. FBG and diabetic successful rate in the groups of the second section

Table 3 showed the diabetic successful rate (DSR) and FBG level of the rats which were fed with high-fat diet combined with STZ 25 mg/kg, 30 mg/kg twice injection. It was observed that the successful rate in 25 mg/kg STZ twice injection group was low (25%). However, the successful rate of 30 mg/kg STZ twice injection group was relatively high (85%), and the number of diabetic rats has been stable until the end of the study. FBG level of DM6 group was also significantly increased compared to control group (*P* < .01).

### 3.4. IPGTT and ITT in the groups of the second section

**Figure 2:**
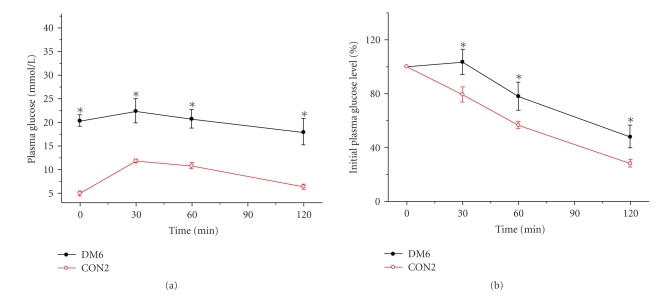
(a) Plasma glucose during intraperitoneal glucose tolerance test (IPGTT) in STZ (30 mg/kg, twice, IP) group (DM6) and control rats after 4 weeks of injection. (b) Percentage of initial glucose level during insulin tolerance test (ITT) in DM6 and control group after 4 weeks of injection. Data shown are means ± SE (*n* = 17–20 rats/group per time point). *P < .01, DM6 versus control, by *t*-test.

**Figure 3:**
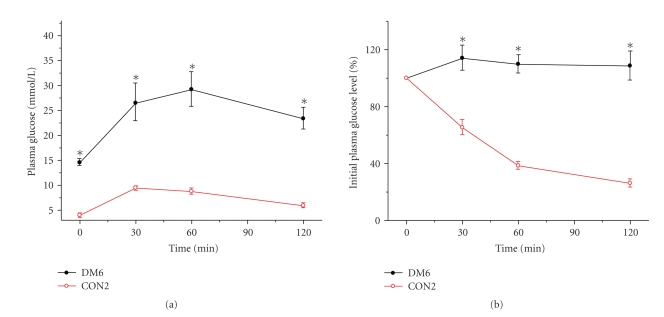
(a) Plasma glucose during intraperitoneal glucose tolerance test (IPGTT) in STZ (30 mg/kg, twice, IP) group (DM6) and control rats after 8 weeks of injection. (b) Percentage of initial glucose level during insulin tolerance test (ITT) in DM6 and control group after 8 weeks injection. Data shown are means ± SE (*n* = 17–20 rats/group per time point). *P < .001, DM6 versus control, by *t*-test.

Since 30 mg/kg STZ twice injection group (DM6) had the higher successful rate and higher FBG level, IPGTT and ITT were performed further in this group at 4 weeks (Figure 2) and 8 weeks (Figure 3) after STZ injection. As shown in Figure 2(a), 4 weeks after STZ injection, the glucose level in DM6 and control group reached the highest level at 30 minutes after glucose injection, and slowly decreased in the following 90 minutes. But the glucose level in DM6 showed hyperglycemia compared to control rats during 120 minutes. The areas under the glucose curves (mmol/L·min) were significantly greater in the STZ injection group compared with controls [(3278 ± 274) mmol/L·min versus (1103 ± 81) mmol/L·min, *P* < .01]. Meanwhile at 8 weeks after STZ injection (Figure 3(a)), the areas under the glucose curves (mmol/L·min) were still significantly greater in the STZ injection group compared with controls [(4498 ± 333) mmol/L·min versus (913 ± 47) mmol/L·min, *P* < .001]. To investigate differences in insulin sensitivity, we performed ITT after 4 weeks (Figure 2(b)) and 8 weeks (Figure 3(b)) STZ injections. We found that, after insulin administration in control group, glucose concentrations declined rapidly; however, the glucose concentrations declined slowly or even not declined in DM6 group within 30 minutes. After 30 minutes, the slopes of these two curves were similar (Figure 2(b)). However, the percentage of initial glucose level in DM6 was shown to be significantly higher than that of control group during 120 minutes. These changes were still significant after 8 weeks STZ injection (Figure 3(b)). This result demonstrated that the rats after STZ 30 mg/kg twice injection presented insulin resistance. All these data indicated that high-fat diet associated with 30 mg/kg STZ twice injection developed a diabetic model which was an analogue to type 2 diabetes mellitus with insulin resistance and hyperglycemia.

### 3.5. Changes of body weight and food intake in 30 mg/kg STZ twice injection group and control groups

According to the data shown above, 30 mg/kg STZ twice injection developed a diabetic rat model with insulin resistance and hyperglycemia. Therefore, we measured the body weight and food intake between this group and the control group. As shown in Table 4, the body weight gain during the study was not statistically different between two groups. Caloric intake of 30 mg/kg STZ twice injection group was significantly higher compared to control group (*P* < .05).

### 3.6. Stability of experimental diabetic model

From the data shown in the second section, 30 mg/kg STZ twice injection might develop a better type 2 diabetic rat model. Therefore, in the third section we investigated the stability of this experimental type 2 diabetic model.

**Figure 4:**
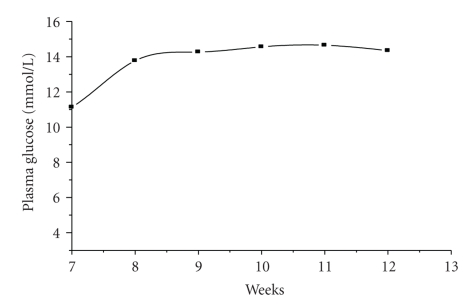
Plasma glucose curve in STZ (30 mg/kg, twice, IP) group (DM6) from 3 weeks after STZ injection to the end of the study (*n* = 17–20 rats).

As shown in Figure 4, the FBG of the model group has been measured every week; this result indicated that the hyperglycemia was stable around 14 mmol/L from 4 weeks after STZ injection to 8 weeks after STZ injection.

The biochemical parameter and ISI at the end of study were presented in Table 5. As can be seen, the high-fat diet group increased the body weight significantly compared with control group; the body weight from STZ injected chow diet rats had no significant difference compared with control group. High-fat diet group presented higher FINS, TG, and TC levels, but there was no significant difference in blood glucose level compared with control groups. FBG, FINS, TG, and TC of HFD-STZ group were significantly increased compared with control group, the ISI was much lower than the control group, which indicated that the insulin sensitivity was remarkably decreased in HFD-STZ group compared to the control group. Moreover, biochemical parameters of the control plus STZ injection group were not significantly changed compared to control group.

### 3.7. Beneficial effect of berberine on the diabetic rats

**Figure 5:**
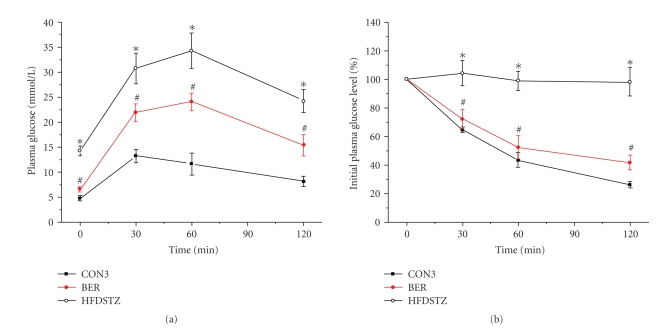
(a) Plasma glucose during intraperitoneal glucose tolerance test (IPGTT) in STZ (30 mg/kg, twice, IP) group (HFD-STZ), berberine trearment group (BER) and control rats (CON3) after 8 weeks injection. (b) Percentage of initial glucose level during insulin tolerance test (ITT) in these three groups after 8 weeks injection. Data shown are means ± SE (*n* = 17–20 rats/group per time point). *P < .01, versus control group, ^*#*^ *P* < .05, versus HFD-STZ group.

Furthermore, we observed whether this animal model was suitable for pharmaceutical research. As shown in Figure 5, Berberine (100 mg/kg) orally administration improved impaired glucose tolerance, and enhanced insulin sensitivity. After 4 weeks of treatment, the areas under the glucose curves (mmol/L·min) were still significantly lower in the berberine treatment group compared with diabetes model group [(2499 ± 167) mmol/L·min versus (3822 ± 344) mmol/L·min, *P* < .05]. In the treatment group, the glucose concentration declined rapidly during ITT. Meanwhile, berberine administration significantly decreased FBG, TC, and TG levels compared to diabetic model group (*P* < .05), the fasting insulin was changed but not significantly, ISI was higher than the model group, and the result indicated that berberine improved insulin sensitivity and abnormal blood lipid. The berberine treatment decreased the body weight slightly but with no significant difference compared with HFD-STZ group (Table 5). This data also demonstrated that the diabetic model we developed might be suitable for pharmaceutical research.

## 4. DISCUSSION

Type 2 diabetes is a complex, heterogeneous, and polygenic disease. There are many underlying factors that contribute to the high blood glucose levels in these type 2 diabetes patients. An important factor is the body's resistance to insulin, essentially ignoring its insulin secretions. A second factor is the falling production of insulin by the *β* cells of the pancreas. Therefore, an individual with type 2 diabetes may have a combination of deficient secretion and action of insulin. Hence, an experimental animal model which aims at mimicking the pathogenesis and clinical feature of human type 2 diabetes mellitus should preferably have these two traits. Among the animal models available, inherited hyperglycaemia and/or obesity in certain strains have been wildly used in the investigations, such as ob/ob mouse, Zucker rats, and OLETF rats. However, those inbred diabetic models are comparatively expensive and not easy to breed. Thus, type 2 diabetic model developed in rodents has been studied for reasons such as short generation time and economic considerations.

Currently, many studies have reported that the high-fat diet (HFD) feeding rats develop insulin resistance [5–7]. At the same time, low-dose STZ has been known to induce a mild impairment of insulin secretion which is similar to the feature of the later stage of type 2 diabetes [1, 2]. Therefore, investigators have started to develop a rat model by high-fat diet following low-dose STZ that would closely mimic the natural history of the disease [1, 2, 4]. Although the appearance of the type 2 diabetes pattern was achieved by combining the feeding of HFD and low dose of STZ treatments in nongenetic, out-bred rats, the injection dose of STZ and its methodologies were not consistent in those studies.

The purpose of the present study was to develop an animal model of type 2 diabetes that would imitate the natural history and the metabolic characteristics of the human syndrome and be responsive to the pharmaceutical treatment. On the other hand, the goal of the present study was to develop an animal model which is neither inherited nor genetically obese, and which is easily accessible, fairly economical, and with high successful rate. The results demonstrated that we had achieved our goals.

The primary attempts of the present study were to identify the dose of STZ that was low enough to develop type 2 diabetes models in HFD rats with higher successful rate and without much insulin deficiency. The different doses of STZ (25, 30, 35, 45 mg/kg, IP) were studied. Injection of STZ (45 mg/kg, IP) after 4 weeks of high-fat diet caused frank hyperglycemia with 100% success rate, which is consistent with literature reports [1]. Further, these rats were insulin sensitive, presenting an insulin-deficient symptom as compared to the control rats. Thus, these fat-fed rats with high dose of STZ (45 mg/kg) were considered resembling type 1 diabetes. In contrast, STZ (30 mg/kg and 35 mg/kg, IP) failed to generate a significant hyperglycemia in HFD-fed rats. Srinivasan et al. have reported the similar results showing that STZ (25 mg/kg, 35 mg/kg, 45 mg/kg, and 55 mg/kg, once injected) could be used to develop diabetic model. Since the dose of STZ (25 mg/kg) did not produce significant hyperglycemia, and fat-fed/STZ (45 and 55 mg/kg, IP) diabetic rats exhibited fairly high glucose and a drastic reduction in the body weights, they finally chose 35 mg/kg STZ injection as the optimum dose, but the successful rate of this method has not been reported [2].

Our second study attempted to find if multiple low doses of STZ could achieve our goal (high successful rate and performance of type 2 diabetes). Multiple low-dose STZ injection to induce diabetes and its complication model have been reported in many studies [9–14]. Multiple low doses of STZ in rats and mice could induce a combination of poisonous and immunological response presenting progressively hyperglycemia. Investigator from Korea has reported that male Sprague-Dawley rats showed rapid chemical destruction of the pancreatic *β* cells when they were given a single high-dose injection of STZ (80 mg/kg, IP); interestingly, multiple low-dose injections of STZ (20 mg/kg for 5 consecutive days, IP) could induce a gradual, autoimmune destruction of *β* cells [13]. Wright Jr. and Lacy reported that rats receiving multiple low doses of STZ (25 mg/kg IP at weekly intervals for 3 weeks) only did not develop diabetes. Immunologic adjuvants played the synergistic role in prompting the induction of diabetes with multiple low doses of STZ in rats [14]. Therefore, it can be proved that multiple low doses of STZ acted to induce a gradual destruction of *β*-cell. This might happen in decompensated phase of type 2 diabetes. High-fat diet has been extensively used to develop insulin resistance [5–7]. Therefore, high-fat diet combined with multiple low dose of STZ might develop a suitable type 2 diabetes animal model which presents not only insulin resistance but also insulin deficiency. The current study proved this point. Our data demonstrated that multiple low doses of STZ (30 mg/kg IP at weekly interval for 2 weeks) produced frank hyperglycemia in HFD-fed rats with highly successful rate, but did not produce the same in regular chow-fed rats. The ISI and ITT all demonstrate the insulin resistance of these diabetic rats. Hence, HFD in combination with multiple low doses of STZ (30 mg/kg, twice injection at weekly interval) can be more considered to characterize the pathophysiology of type 2 diabetes.

Furthermore, the diabetic model we developed produces hyperglycemia around 14.5 mmol/L, which is reasonable to be treated by therapeutic compound as practiced clinically. Therapeutically, it is difficult to reduce elevated blood glucose except for administration of insulin. Berberine, the major active constituent of Rhizoma coptidis, is used clinically in the treatment of diarrhea as an antimicrobial agent. Early as 1986, investigators from china have started to report the hypoglycemic effect of berberine. In 1999, Yuan reported that berberine exerted beneficial effect on the treatment of diabetes clinically; other investigators also proved its role in the treatment of type 2 diabetes in clinic [15–18]. The mechanisms of the antidiabetic effect of berberine involved multiple factors. Yin et al. reported that berberine improved glucose metabolism through induction of glycolysis in many cell lines including 3T3-L1 adipocytes, L6 myotubes, C2C12 myotubes, and H4IIE hepatocytes, which might be related to inhibition of glucose oxidation in mitochondria [19, 20]. Others revealed that the underlying mechanism for berberine improves insulin resistance and lowers blood sugar possibly through activating the AMP-activated protein kinase (AMPK) pathway [21]. It has also been demonstrated that inhibiting phosphorylation of IKK*β* might be a cofactor of berberine in achieving its anti-inflammation and insulin-resistance-improving effects [22]. The latest study also proved that berberine could inhibit fructose-induced insulin resistance in rats possibly by increasing the expression HNF-4*α* in liver [23]. HNF-4*α* is a positive regulator of PEPCK so an increase of HNF-4*α* would result in increased gluconeogenesis. Hence, we administrated berberine as antidiabetic drug in the present study, and our result also demonstrated that berberine treatment could decrease the insulin resistance and improve impaired glucose tolerance. Meanwhile, we also found that berberine could correct lipid metabolism disorders, which indicated that the treatment of berberine might play an important role in diabetic complication. Altogether, the diabetic model we developed was suitable to investigate not only the pathogenesis but also the pharmaceutical selection. Meanwhile, the current study considered the incidence of diabetes in the group. This method will successfully produce type 2 diabetic rat models, as well as provide the enough number of diabetic rats once which will make the investigation more convincing.

Conclusively, our study demonstrates that a combination of HFD and multiple low doses of STZ injection could be effectively used to generate a rat model that mimics the natural history and metabolic characteristics of type 2 diabetes in humans. It was also useful in evaluating the effect of therapeutic compounds on the treatment of type 2 diabetes.

## Figures and Tables

**Table 1:** The diabetic successful rate and FBG level in the groups of the first section.

GROUP	*N*	*N*1	FBG (mmol/L)	DSR
CON1	20	—	5.18 ± 0.55	—
DM1	20	2	5.54 ± 0.99	10%
DM2	20	7	7.92 ± 1.37	35%
DM3	20	8	8.48 ± 1.53	40%
DM4	20	20	24.5 ± 3.75*	100%

*N*: number of rats in each group; *N*1: number of rats with FBG > 7.8 mmol/L which were injected with STZ after 8 weeks, CON1: control group; DM1: STZ 25 mg/kg IP; DM2: STZ 30 mg/kg IP; DM3: STZ 35 mg/kg IP; DM4: STZ 45 mg/kg IP; FBG: fasting blood glucose; DSR: diabetic successful rate. *P < .001 versus control group.

**Table 2:** FBG, FINS, and ISI in STZ (45 mg/kg, IP) group and control group.

Group	*N*	FBG (mmol/L)	FINS (mIU/L)	ISI
CON1	20	5.18 ± 0.55	11.8 ± 2.93	−3.85 ± 0.27
DM4	20	24.5 ± 3.75**	3.97 ± 0.86*	−4.10 ± 0.32

CON1: control group; DM4: 8 weeks after STZ 45 mg/kg IP once, *N*: number of rats in each group; FBG: fasting blood glucose at 8 weeks after STZ injection; ISI = ln (FBG × FINS)^−1^; FINS: fasting plasma insulin level; **P < .001, *P < .05, DM4 versus control group.

**Table 3:** The diabetic successful rate and FBG level in the groups of the second section.

Group	*N*	*N*1	*N*2	*N*3	*N*4	*N*5	FBG (mmol/L)	DSR
CON2	20	—	—	—	—	—	4.27 ± 0.41	—
DM5	20	0	2	3	5	5	5.93 ± 0.93	25%
DM6	20	4	10	12	17	17	14.26 ± 0.57*	85%

*N*: number of rats in the group; *N*1: number of rats with FBG > 7.8 mmol/L at one week after once STZ injection; *N*2: number of rats with FBG > 7.8 mmol/L at one week after twice STZ injection; *N*3: number of rats with FBG > 7.8 mmol/L at two weeks after twice STZ injection; *N*4: number of rats with FBG > 7.8 mmol/L at three weeks after twice STZ injection; *N*5: number of rats with FBG > 7.8 mmol/L at four weeks after twice STZ injection; FBG: fasting blood glucose; DSR: diabetic successful rate; CON2: control group; DM5: twice STZ 25 mg/kg IP group; DM6: twice STZ 30 mg/kg IP group; *P < .01 versus control group.

**Table 4:** Body weight and food intake of control group and DM6.

Week	Body weight (g)		Food intake (KJ/d)	
	CON2	DM6	CON2	DM6
1	272.3 ± 4.9	272.5 ± 5.9	54.5 ± 1.0	73.6 ± 1.1*
2	291.6 ± 5.5	294.3 ± 6.9	48.2 ± 1.2	65.4 ± 2.2*
3	294.4 ± 6.3	296.9 ± 7.4	47.7 ± 0.7	59.3 ± 0.7*
4	300.6 ± 5.8	304.4 ± 7.7	48.8 ± 1.5	63.1 ± 1.3*
5	306.4 ± 7.1	314.9 ± 7.1	53.4 ± 1.3	67.5 ± 1.2*
6	329.4 ± 7.5	334.2 ± 9.8	54.9 ± 1.9	64.1 ± 1.7*
7	356.2 ± 8.1	363.1 ± 9.5	51.9 ± 1.0	67.0 ± 0.6*
8	374 ± 9.0	373.0 ± 9.4	51.7 ± 1.0	69.4 ± 1.0*
9	384.8 ± 8.9	383.6 ± 10.1	58.6 ± 1.7	73.4 ± 4.2*
10	385.2 ± 8.9	379.2 ± 11.0	60.9 ± 2.2	91.4 ± 2.7*
11	389.9 ± 10.0	375.6 ± 10.1	69.2 ± 1.5	95.3 ± 4.0*
12	415.6 ± 11.8	382.1 ± 17.3	72.3 ± 3.6	99.5 ± 3.0*

CON2: control group; DM6: high-fat diet with STZ 30 mg/kg twice injection group. Values are means ± SE; *N*: number of rats, *P < .05 versus control group.

**Table 5:** The biochemical parameter and ISI in the groups of the third section.

Group	CON3	C-STZ	HFD	HFD-STZ	BER
*N*	20	20	20	17	17
Body weight (g)	415.6 ± 11.8	397.9 ± 14.2	477.3 ± 23.1*Δ	382.1 ± 17.3	368.6 ± 19.8
FBG (mmol/L)	4.49 ± 0.41	5.25 ± 0.44	5.56 ± 0.52	14.79 ± 0.32**	6.48 ± 0.56Δ
FINS (mU/L)	11.03 ± 1.68	12.04 ± 0.26	19.64 ± 6.83*	9.17 ± 1.34	10.04 ± 0.91
ISI	−3.87 ± 0.15	−3.93 ± 0.20	−4.69 ± 0.21*	−4.87 ± 0.15*	−3.68 ± 0.09Δ
TG (mmol/L)	0.66 ± 0.09	0.63 ± 0.08	1.91 ± 0.33**	1.70 ± 0.21**	0.96 ± 0.17*Δ
TC (mmol/L)	1.72 ± 0.11	1.78 ± 0.19	3.35 ± 0.26**	2.99 ± 0.53**	2.67 ± 0.58*

CON3: control group; C-STZ: regular chow feed group with STZ 30 mg/kg twice IP; HFD: high-fat diet group; HFD-STZ: model group by high-fat diet with STZ 30 mg/kg twice IP; BER: berberine treatment group; FBG: fasting blood glucose; FINS: fasting blood insulin; ISI = ln(FBG × FINS)^−1^; TG: triglyceride; TC: total cholesterol. Values are means ± SE, **P < .01, *P < .05 versus control group, Δ*P* < .05 versus HFD-STZ group.
